# A Representation of Metastatic Plasma Cell Myeloma as an Uncommonly Shaped Liver Tumor—A Case Report

**DOI:** 10.3390/medicina60081237

**Published:** 2024-07-30

**Authors:** Tomasz Skołozdrzy, Jan Wojciechowski, Mateusz Gural, Agata Kaniewska, Maciej Miernik, Maciej Romanowski

**Affiliations:** Department of General and Oncological Surgery, Pomeranian Medical University, Ul. Unii Lubelskiej 1, 71-252 Szczecin, Poland

**Keywords:** plasma cell myeloma, uncommon shape, metastasis, liver

## Abstract

The presence of an oval-shaped lesion in the liver is mainly associated with either primary liver cancer or metastatic disease from another malignancy. However, we present the case of a 62-year-old patient diagnosed with plasma cell myeloma, which reveals that these kinds of lesions can also be found during the course of this disease. Rarity and non-specificity make this a very challenging diagnosis for radiologists. It involves a special alert from the doctors taking care of the patient. Biopsy may sometimes be necessary to make a correct diagnosis. It is significant to ensure that the correct treatment is implemented and that the patient is not exposed to the unnecessary diagnosis of another neoplastic disease.

## 1. Introduction

Multiple myeloma is the second most common hematological malignancy [1]. It represents approximately 1.8% of all new cancer cases diagnosed each year in the United States. In Europe, it is the third most common blood cancer (10–15% of all blood cancers) and represents about 1% of all cancers [2]. The exact etiology is unknown; however, frequent alterations and translocations in the promoter genes, especially on chromosome 14, are often found in it, and it is thought that they have an influence in disease development [3]. Alcohol consumption, obesity, insecticides, and radiation exposure could also contribute to this disease’s occurrence [3].

Multiple myeloma arises from monoclonal gammopathy of undetermined significance (MGUS). This condition is defined as the presence of monoclonal immunoglobulins in the blood (30 g/L of M protein, less than 10% of clonal bone marrow plasma) or urine without end-organ damage. It is asymptomatic and occurs in over 3% of people above the age of 50. 

In some patients, in addition to MGUS and multiple myeloma, another pre-malignant state is distinguished: smoldering multiple myeloma. The following must be stated in order to diagnose this condition: serum monoclonal protein (IgG or IgA) ≥ 3 gm/dL or urinary monoclonal protein ≥500 mg per 24 h and/or clonal bone marrow plasma cells from 10% to 60% without myeloma-defining events or amyloidosis [4]. The median time for progression from SMM to MM is 5 years and depends on the proportion of plasma cells in the bone marrow and the serum monoclonal protein level at diagnosis [1].

Multiple myeloma’s most common events are included in the CRAB acronym. It consists of hypercalcemia, anemia, renal insufficiency, and bone lesions. To add on to these, these events are usually accompanied by hyperviscosity, amyloidosis, fatigue, and recurrent infections [1]. In one retrospective study, the following symptoms of newly diagnosed multiple myeloma were most common: anemia (73%), bone pain (58%), elevated creatinine (48%), fatigue (32%), hypercalcemia (28%), and weight loss (24%) [5].

The diagnosis of multiple myeloma includes ≥10 clonal bone marrow plasma cells or a biopsy-proven plasmacytoma, as well as evidence of one or more defining events: CRAB attributable to the plasma cell disorder, bone marrow clonal plasmacytosis ≥ 60% serum involved/uninvolved free light chain ratio ≥ 100, or >1 focal lesion on magnetic resonance imaging [4].

Differential diagnosis of multiple myeloma includes monoclonal gammopathy of undetermined significance (MGUS), amyloidosis, Waldenstrom macroglobulinemia, solitary plasmacytoma, and smoldering multiple myeloma [3].

Multiple myeloma is mainly associated with bone lesions; however, an extramedullary disease can be found in other organs like the liver. The presence of lesions in the liver in living patients is very rare, and if it happens, it is usually in the form of diffuse plasma cells. Therefore, the appearance of a singular, oval-shaped tumor in the liver in the case of a patient with a history of multiple myeloma could be confusing and suggest the presence of another malignancy. In this case report, we present a 62-year-old patient with a liver tumor which happened to be a solitary liver plasmacytoma in the course of multiple myeloma. 

## 2. Case Description

A 62-year-old female patient diagnosed with plasma cell myeloma IgG kappa II A (according to Durie-Salmon, R-ISS II) was referred to the Department of General and Oncological Surgery for percutaneous liver biopsy due to the detection of a hypermetabolic lesion in the liver during follow-up PET/CT confirmed by contrast-enhanced MRI of the abdomen. Due to her underlying disease, the patient was treated in the past with many cycles of combination chemotherapy, two bone marrow auto-transplants, thalidomide maintenance therapy, and bone radiotherapy. 

Due to the detection of a hypermetabolic lesion in the liver in the follow-up PET/CT scan (lesion 35 mm SUV max 6.1 in the right lobe), the decision was made to perform an abdominal MRI with contrast, which confirmed the presence of a lesion in segment 6 of the liver, 17 mm in size, with a moderately increased signal at T2 and T2fs, a decreased signal at T1 with weak heterogeneous contrast enhancement, and limiting diffusion of a non-characteristic lesion, more suggestive of a malignant lesion. Observation in the form of regular imaging studies was decided upon. Over the following months, a gradual regression of the lesion size to a maximum of 10 mm was observed, followed by a new increase up to 57 × 46 mm.

**Figure 1:**
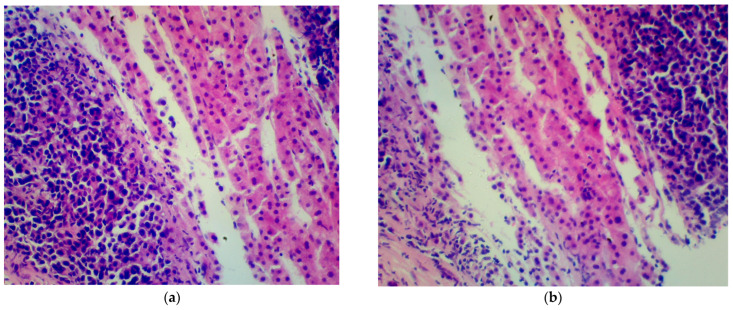
Histopathological examination of hepatic tissue (hematoxylin-eosin-stained histological images). Small, dark cells of plasma cell myeloma can be seen (**a**,**b**).

A percutaneous liver biopsy was performed (Figure 1). The histopathological examination described a hepatic tissue with infiltration of plasma cell myeloma. IHC: CD38+, CD138+, chromogranin−, synaptophysin−, and Ki67+ in approximately 80% of cells. The results of the biopsy confirmed that the lesion, which seemed to be an independent tumor, was in fact the outcome of the underlying disease.

**Figure 2:**
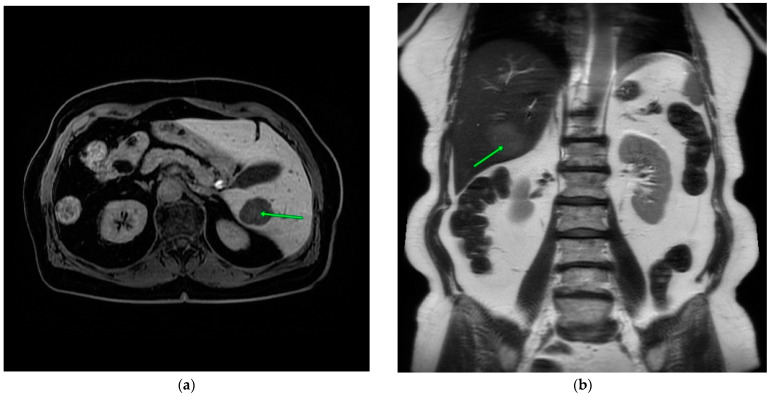
Abdominal MRI. Green arrows point the lesion (**a**,**b**).

An additional CT scan was performed after the biopsy, which peripherally showed a single, fairly well-demarcated oval nodular mass measuring approximately 57 × 46 mm with strong heterogeneous enhancement, and a relatively rapid washout in the venous/delayed phase was detected in segment VI, causing peripheral perfusion abnormalities and accentuation of the intrahepatic bile ducts (Figure 2a). The lesion was vascularized by a prominent branch of the right hepatic artery (Figure 2b).

After discovering a plasmacytoma of the liver, the Hematology Clinic changed the chemotherapy type. After receiving the cardiologist’s consent, the previous chemotherapy type was terminated. The patient started a KRD therapy protocol (carfilzomib, lenalidomide, dexamethasone) with the exclusion of a cytostatic treatment and is currently being treated in the clinic.

## 3. Discussion

Multiple myeloma’s most common complications involve bone loss, hypercalcemia, anemia, leukopenia, renal failure, severe pain, and thrombocytopenia [6]. Myeloma cells proliferate in bone marrow and circulate through the bloodstream. Due to circulation through lymphatics and the reticuloendothelial system spleen, lymph node or liver infiltrations are possible [7]. The involvement of the gastrointestinal system in patients with multiple myeloma is a rare event, only described in the literature in individual case reports. Liver involvement through multiple myeloma is usually a terminal anomaly, uncommon in living patients [8,9]. However, it is observed in around 30–40% of autopsy cases. Compared to other hematological malignancies, this is quite a low number; liver involvement is found in 80–100% of cases of chronic leukemia and myeloproliferative disease, 60–70% of cases of acute leukemia, and 50–60% of cases of non-Hodgkin’s lymphoma [7]. 

Perez-Soler et al. [10] carried out an autopsy series of 128 cases with multiple myeloma. In total, 21 patients had liver tissue involvement, and in 10 of them, the autopsy showed diffuse infiltration of the liver by plasma cells. Liver involvement can lead to a range of symptoms. Clinical manifestations in the case of liver involvement are nonspecific. They include hepatomegaly, splenomegaly, jaundice, and ascites [11]. A study conducted by Thomas et al. [12] showed that 58% of patients had hepatomegaly described as percussible dullness of more than 12 centimeters or a palpable liver edge 4 centimeters below the right costal margin. Overall, 25% of patients had splenomegaly, while 14% had jaundice, with serum bilirubin values ranging from 3.2 to 17.3 mg/dL. Only 9% of patients had a completely normal liver on pathological examination. On physical examination, our patient did not present with hepato- or splenomegaly, and laboratory tests did not detect elevated bilirubin or liver enzyme levels. However, in multiple myeloma patients, elevations in liver function tests should not be ignored. 

The histological pattern of liver involvement in multiple myeloma might be in the form of extramedullary plasmacytoma, light chain deposition disease, amyloidosis, or a diffuse infiltrative pattern. Massive liver involvement can either be from diffuse sinusoidal flooding or tumor-forming plasmacytomas [13]. The latter, which happened in our patient’s case, is extremely rare, with reported rates ranging from 0.3% to 3% [9]. In 2006, Talamo et al. reported 9 cases of nodular liver infiltration in a cohort of 2584 patients with multiple myeloma [14]. In 2015, Huang et al. [15] performed a literature review which summarized all 13 available studies describing multiple myeloma with nodular hepatic involvement. Only two of the reported cases had solitary nodular liver lesions, just like in our patient’s case, with the remaining cases presenting with multiple lesions. Contrary to our study, liver involvement was also common, with eight of the cases manifesting with abdominal pain and/or other gastrointestinal symptoms. Overall, 6 out of 13 cases proceeded with abnormalities in liver function tests, with alkaline phosphatase most commonly being elevated, and in 3 cases, liver test functions were not reported. In our patient’s case, baseline laboratory findings showed no elevation in alanine aminotransferase (ALT), aspartate aminotransferase (AST), alkaline phosphatase (ALP) or gamma-glutamyltransferase (GGTP). In the review, the distribution of the monoclonal protein was varied. The most common monoclonal component was IgG kappa (*n* = 4), as happened in our case. However, it cannot be said that its presence is characteristic of patients with liver plasmacytoma in the course of multiple myeloma, as in the review, lambda light chain (*n* = 3) and IgA kappa (*n* = 3) occurred only slightly less often. 

This systematic review shows that asymptomatic plasmocytoma of the liver in the course of multiple myeloma is a very rare finding that needs a comprehensive approach involving multiple specialties. Radiological imaging, histopathological examinations of tissue samples from surgical biopsies and using immunohistochemical analysis are pivotal. Combining these diagnostic methods implements a profound understanding of the tumor and guarantees that such a rare finding is accurately diagnosed. 

Differential diagnoses in liver plasmacytoma include hepatocellular carcinoma, hepatic adenoma and hypervascular metastases including melanoma, neuroendocrine tumors, renal cell carcinoma, and thyroid carcinoma [11]. Similarities between lesions in multiple myeloma and hepatocellular carcinoma may be challenging for a distinction.

The mechanism of hepatic involvement in multiple myeloma is unclear and can be a sign of an advanced-stage disease. Extramedullary lesions happen when the clonal plasma cells lose their dependence on the BM milieu for growth. Research shows that incidence is higher in patients after allogenic stem cell transplantation [16]. In our patient’s case, she was treated with two bone marrow autotransplants. Our patient was also treated with thalidomide, and liver involvement during thalidomide therapy was reported in the literature previously. However, in this case, adhesion molecules were not present in liver infiltration, contrary to our patient’s case [17]. Our patient was treated with thalidomide for two years 10 years ago, so the time span is probably too long to identify this therapy as a cause of liver plasmacytoma.

Using cytogenetic and molecular tests is important in making a diagnosis. Karyotyping and FISH (fluorescence in situ hybridization) are the methods used in identifying chromosomal aberrations. Research shows that FISH is a much more efficient method; however, combining both of them can provide better information than using one test alone. Based on cytogenetic testing, hyperdiploid multiple myeloma (H-MM) and on-hyperdiploid multiple myeloma (NH-MM) were identified. Hyperdiploidia is more common in older patients and is usually less aggressive. Worse prognosis is characteristic for hypodiploidia, especially that involving chromosomal arm deletions. The International Myeloma Working group recommends genetic diagnostics, especially using the FISH method in every patient with multiple myeloma diagnosis in order to identify high-risk patients [18,19]. High-risk multiple myeloma includes any of the following cytogenetic aberrations: t(14:16), t(14:20), del17p13, t(4:14), and 1q gain. Standard risk involves trisomies, t(11:14), and or t(6:14).

There are several staging systems of multiple myeloma. Currently, the most popular is the Revised International Staging System (R-ISS) due to its simplicity and robust prognostic information that it gives. It involves β_2_ microglobulin, albumin, lactate dehydrogenase (LDH) levels and cytogenetics, and it is as follows:

- Stage 1—β_2_ microglobulin less than 3.5 mg/L, albumin level greater than or equal to 3.5 g/dL, normal LDH, and standard risk cytogenetics.
- Stage 2—Neither stage 1 nor stage 3.
- Stage 3—β_2_ microglobulin level greater than 5.5 mg/L and high-risk cytogenetics (del(17p) and/or t(4:14), and/or t(14:16) or elevated LDH) [20].

R-ISS classification overshadowed the previous gold standard of staging, the Durie–Salmon system. It included tumor cell mass, hemoglobin, calcium, IgA and IgG levels, urine monoclonal protein levels, and the extent of bone damage identified via X-rays. Just like R-ISS classification, it divided patients into three stages; however, it sub-grouped them further into groups A and B based on renal function measured by serum creatinine level [3,21].

Staging classification is helpful in estimating the five-year overall survival (OS) and progression-free survival (PFS). For example, in R-ISS, five-year OS is 82% and PFS was 55%. Stage II of the disease has a five-year OS of 62% and PGS of 36%. Stage III is characterized by a five-year OS of 40% and PFS of 24% [17]. High-risk cytogenetic abnormalities are also important in calculating outcomes; in particular, t(4:14), t(14:16), f(14:20) and 17p deletions are known to decrease OS [3,22].

Imaging plays a crucial role in diagnosing multiple myeloma. Conventional skeletal radiography has been traditionally used to identify bone disease in patients, and to this day, it is a recommended first-line imaging method in the diagnosis and staging of this disease [15]. Newer and more accurate imaging methods include computed tomography (CT), magnetic resonance imaging (MRI), and 18F-fluorodeoxyglucose PET with CT (PET-CT). CT and MRI are used in order to further evaluate lesions that are unclear on plain radiography, assess the nature and extent of soft tissue disease, evaluate spinal cord compression, and differentiate between malignant and benign vertebral compression fractures. PET-CT and MRI are also frequently used in patients with asymptomatic multiple myeloma or plasmacytoma, as they can detect infiltrative processes of the bone marrow in the absence of bone lesions [15].

Liver involvement in the course of multiple myeloma can present differently based on the used imaging technique. In ultrasonography, the nodular form presents a well-defined hypoechoic nodule with or without a target. In a CT scan, they are hypodense. In an MRI, they are hypointense or hyperintense on T1-weighted images and hyperintense on T2-weighted images. In both a CT scan and an MRI, they can be enhanced or non-enhanced. In several cases, they show arterial enhancement and delayed washout [11]. In the systematic review by Huang et al. [15], out of 13 cases, CT was used in 10 cases, ultrasonography was used in 8 cases, and in 3 cases, MRI and PET were used to diagnose the disease.

Percutaneous liver biopsy is crucial in order to histologically differentiate the pathology. Using it enables establishing a diagnosis, assessing prognosis and staging, and thus gives a clue about treatment options and clinical management. The procedure is relatively safe with a great diagnostic yield. Due to the help of advanced imaging techniques like ultrasound, computed tomography, and magnetic resonance imaging, the biopsy can be accurately targeted towards the tumor, which decreases the rate of complications.

Complications are rare, with the most common being pain located at the biopsy site or in the right shoulder due to subcapsular swelling or bleeding. Less common complications include transient hypotension, hemorrhaging, a pneumothorax, biliary peritonitis, transient bacteremia, portal vein thrombosis, or a subphrenic abscess [23].

The symptomatic form of multiple myeloma is an indication for treatment. The choice depends on the patient’s eligibility for an autologous stem cell transplant (ASCT). The patients are divided into two categories—high- or standard-risk multiple myeloma. The patients eligible for an ASCT receive induction chemotherapy in order to decrease the tumor size. This usually lasts for four to six cycles administered within 3 to 4 months. For high-risk-patients who are transplant-eligible, typical induction therapy includes four cycles of daratumumab, bortezomib, lenalidomide, and dexamethasone. For standard-risk patients with transplantation eligibility, four cycles of bortezomib, lenalidomide, and dexamethasone (VRd) are used. Meanwhile, 8 to 12 cycles of this scheme can also be used in high-risk transplant-ineligible patients followed by maintenance bortezomib therapy [3]. Supportive treatment in multiple myeloma includes the treatment of complications like nephropathy, thrombosis, anemia, hypercalcemia, or skeletal lesions. The use of radiotherapy is limited to local changes, especially osseous plasmacytomas, pain treatment, pathological fractures, or spinal cord compression syndrome [24].

In the presence of hepatic involvement, as in our case, the disease usually requires systemic therapies; however, choosing the approach depends on the presence and intensification of liver dysfunction. Due to the rarity and molecular and proliferative heterogeneity of this condition, no guidelines exist [8]. In the case of our patient, liver involvement was found over ten years after her multiple myeloma diagnosis. She underwent many treatments in the past, including CTD, VTD, DVD, complementary treatment with thalidomide, two bone marrow auto-transplants, and bone radiotherapy. Therefore, in her case, multiple myeloma is a chronic disease, resistant to treatment. KRd scheme was implemented because in research, this is the scheme that gives the best effect in patients with treatment-resistant MM.

## 4. Conclusions

In conclusion, single plasmacytoma of the liver in patients with multiple myeloma is a very rare occurrence. This condition’s rarity and non-specificity make it a very challenging diagnosis for radiologists. Using biopsy is crucial in identifying such rare diseases. Liver enzyme elevation or non-obstructive jaundice in patients with multiple myeloma is an alarming sign.

## Data Availability

The original contributions presented in this study are included in the article; further inquiries can be directed to the corresponding author.

## References

[1] Yang P., Qu Y., Wang M., Chu B., Chen W., Zheng Y., Niu T., Qian Z. (2022). Pathogenesis and Treatment of Multiple Myeloma. MedComm.

[2] Waszczuk-Gajda A., Szafraniec-Buryło S., Kraj L., Skwierawska K., Aleksandrowicz K., Basak G., Brzozowska M., Wierzba W., Jędrzejczak W.W., Śliwczyński A. (2023). Epidemiology of Multiple Myeloma in Poland in the Years 2008–2017. Arch. Med. Sci..

[3] Albagoush S.A., Azevedo A.M. Cancer, Multiple Myeloma. Nih.gov. https://www.ncbi.nlm.nih.gov/books/NBK534764/.

[4] Rajkumar S.V. (2022). Multiple Myeloma: 2022 Update on Diagnosis, Risk Stratification, and Management. Am. J. Hematol..

[5] Kyle R.A., Gertz M.A., Witzig T.E., Lust J.A., Lacy M.Q., Dispenzieri A., Fonseca R., Rajkumar S.V., Offord J.R., Larson D.R. (2003). Review of 1027 Patients with Newly Diagnosed Multiple Myeloma. Mayo Clin. Proc..

[6] Vrtis M.C. (2024). Multiple Myeloma. Home Healthc. Now.

[7] Wu X.-N., Zhao X.-Y., Jia J.-D. (2009). Nodular Liver Lesions Involving Multiple Myeloma: A Case Report and Literature Review. World J. Gastroenterol. WJG.

[8] Singh M., Singh H., Pham P., Rizvi B., Rao R. (2021). Extramedullary Multiple Myeloma with Hepatic Involvement. Cureus.

[9] Harada N. (2024). Multiple Myeloma Presenting as Nodular Hepatic Lesions Mimicking Hepatocellular Carcinoma. Int. J. Hematol..

[10] Perez-Soler R., Esteban R., Allende E., Saloma C.T., Julia A., Guardia J. (1985). Liver involvement in multiple myeloma. Am. J. Hematol..

[11] Han Y.J., Park M. (2023). Hypervascular Nodular Hepatic Involvement in Multiple Myeloma: A Case Report. J. Clin. Imaging Sci..

[12] Thomas F.B., Clausen K.P., Greenberger N.J. (1973). Liver Disease in Multiple Myeloma. Arch. Intern. Med..

[13] Rahhal F.E., Schade R.R., Nayak A., Coleman T.A. (2009). Hepatic Failure Caused by Plasma Cell Infiltration in Multiple Myeloma. World J. Gastroenterol..

[14] Talamo G., Cavallo F., Zangari M., Barlogie B., Lee C.-K., Pineda-Roman M., Kiwan E., Krishna S., Tricot G. (2006). Clinical and Biological Features of Multiple Myeloma Involving the Gastrointestinal System. Haematologica.

[15] Huang H., Bazerbachi F., Mesa H., Gupta P. (2015). Asymptomatic Multiple Myeloma Presenting as a Nodular Hepatic Lesion: A Case Report and Review of the Literature. Ochsner J..

[16] Ghobrial I.M. (2012). Myeloma as a model for the process of metastasis: Implications for therapy. Blood.

[17] Nozza A., Castagna L., Rahal D., Magagnoli M. (2003). Massive liver involvement in a patient with multiple myeloma during Thalidomide treatment. Haema.

[18] Sommaluan S., Rerkamnuaychoke B., Pauwilai T., Chancharunee S., Onsod P., Pornsarayuth P., Chareonsirisuthigul T., Tammachote R., Siriboonpiputtana T. (2017). The Utilization of Karyotyping, iFISH, and MLPA for the Detection of Recurrence Genetic Aberrations in Multiple Myeloma. Asian Pac. J. Cancer Prev..

[19] Fonseca R., Bergsagel P.L., Drach J., Shaughnessy J., Gutierrez N., Stewart A.K., Morgan G., Van Ness B., Chesi M., Minvielle S. (2009). International Myeloma Working Groupmolecular classification of multiple myeloma: Spotlight review. Leukemia.

[20] Palumbo A., Avet-Loiseau H., Oliva S., Lokhorst H.M., Goldschmidt H., Rosinol L., Richardson P., Caltagirone S., Lahuerta J.J., Facon T. (2015). Revised International Staging System for Multiple Myeloma: A Report from International Myeloma Working Group. J. Clin. Oncol..

[21] Deng S., Zhang B., Zhou Y., Xu X., Li J., Sang S., Zhang W. (2018). The Role of 18F-FDG PET/CT in Multiple Myeloma Staging according to IMPeTUs: Comparison of the Durie–Salmon plus and Other Staging Systems. Contrast Media Mol. Imaging.

[22] Goldman-Mazur S., Jurczyszyn A., Castillo J.J., Waszczuk-Gajda A., Grząśko N., Radocha J., Bittrich M., Kortüm K.M., Gozzetti A., Usnarska-Zubkiewicz L. (2020). A Multicenter Retrospective Study of 223 Patients with T(14;16) in Multiple Myeloma. Am. J. Hematol..

[23] Chan M., Navarro V.J. Percutaneous Liver Biopsy. PubMed. https://www.ncbi.nlm.nih.gov/books/NBK553146/.

[24] Ailawadhi S., Frank R., Ailawadhi M., Kanji Z., Jani P., Fiala M., Abdulazeez M., Ahmed S., Aggarwal C.S., Aulakh S. (2021). Utilization of Radiation Therapy in Multiple Myeloma: Trends and Changes in Practice. Ann. Hematol..

